# Visible light functioning photocatalyst based on Al_2_O_3_ doped Mn_3_O_4_ nanomaterial for the degradation of organic toxin

**DOI:** 10.1186/s11671-015-0990-4

**Published:** 2015-09-09

**Authors:** Safi Asim Bin Asif, Sher Bahadar Khan, Abdullah M Asiri

**Affiliations:** Chemistry Department, Faculty of Science, King Abdulaziz University, P. O. Box 80203, Jeddah, 21589 Saudi Arabia; Center of Excellence for Advanced Materials Research, King Abdulaziz University, P.O. Box 80203, Jeddah, 21589 Saudi Arabia

**Keywords:** Al_2_O_3_ doped Mn_3_O_4_, Nanomaterial, Brilliant cresyl blue, Organic pollutant, Solar photocatalyst

## Abstract

Al_2_O_3_ doped Mn_3_O_4_ nanomaterial was synthesized by low-temperature stirring method and applied as a catalyst for the degradation of organic pollutants under solar light for prospective environmental applications. The morphological and physiochemical structure of the synthesized solar photocatalyst was investigated by X-ray diffraction (XRD), field emission scanning electron microscopy (FESEM), energy-dispersive spectroscopy (EDS), Fourier transform infrared spectroscopy (FT-IR), and X-ray photoelectron spectroscopy (XPS). FESEM showed a mixture of nanowires and aggregated nanoparticles. This Al_2_O_3_ doped Mn_3_O_4_ nanomaterial exhibited high solar photocatalytic degradation in a short time when applied to brilliant cresyl blue (BCB). Thus, the synthesized nanoparticles can be used as an efficient solar photocatalyst for the degradation of BCB.

## Background

Recent industrial development has improved living standards but due to insufficient environmental monitoring, the continuous discharge of massive industrial pollutants (organic compounds, trace metals, etc.) is creating serious environmental problems. Since these pollutants are carcinogenic at trace levels for aquatic and non-aquatic organisms, a wide range of techniques including physical, chemical, and biological methods have been developed for water treatment [1–5]. Environmental degradation due to various types of pollutants has led to global interest in the vital and robust technology known as “nanotechnology.”

In particular, the photocatalytic oxidation process using heterogeneous photocatalysis is regarded as a promising technology to decompose harmful pollutants into final non-toxic products [6–8]. However, most photocatalysts can only be activated under UV-light irradiation because of their large band gap, resulting in low photo-electronic transition efficiency since the ultraviolet light represents only 4 % of the solar spectrum [9]. Therefore, it is necessary and desirable to develop visible light-driven photocatalysts with high efficiency for the degradation of environmental pollutants [7].

Nanostructured transition metal oxides have been considered important materials because of their electronic, optical, photonic, and catalytic properties. Their size reduction to nanoscale can effectively change their physical and chemical properties and specifically improve their potential [3]. Since they have efficient EMR absorption in the visible region, many metal oxides and doped metal oxides have been recommended for the photocatalytic degradation of organic pollutants. Photocatalytic degradation of azo dye in water was effectively carried out with ZnO. Similarly, acid red B dye was degraded by using TiO_2_. Further studies reported the photocatalytic degradation of methyl orange by zinc ferrite-doped titania. Semiconductor iron (II) oxide has also been researched in photocatalytic bleaching of dyes. Furthermore, a large number of other systems such as transition metal-doped TiO_2_ and nitrogen-doped TiO_2_ have been utilized for the photosensitization of dyes [8].

Manganese oxides have been extensively studied as a well-known transition metal oxide because of their unique chemical and physical properties. Manganese oxides have potential application in ion sieves, molecular sieves, catalysis, cathode materials for secondary rechargeable batteries, super capacitors, and new magnetic materials. Research has been reported into manganese oxide nanomaterials with different morphologies, such as nanorods, nanowires, nanotubes, and urchin-like nanostructure [2].

Manganese oxides have been used as one of the most promising electrode materials for super capacitor applications with respect to their natural abundance, low cost, environmentally friendly nature, wide voltage window, and high specific capacitance [5]. More specifically, the performance of manganese oxides dispersed in silica and alumina has been explored in catalytic ozonation of acetone by Oyama and colleagues [1].

In the research reported in this article, we synthesized Al_2_O_3_ doped Mn_3_O_4_ nanomaterial (NM) and characterized it by FESEM, EDS, XRD, XPS, FT-IR, and UV-visible. Further, we evaluated the photocatalytic performance of ΝΜ at different pH under solar light, using brilliant cresyl blue (BCB) as organic pollutant.

## Methods

### Materials

Analytical grade chemicals including aluminum nitrate nonahydrate (Al(NO_3_)_3_ · 9H_2_O), manganese nitrate tetrahydrate (Mn(NO_3_)_2_ · 4H_2_O) (used as precursors of Al_2_O_3_ doped Mn_3_O_4_ multi-metal oxide nanoparticles), BCB, sodium hydroxide NaOH, and 99 % pure ethanol were purchased from Sigma-Aldrich.

### Synthesis of Al_2_O_3_ doped Mn_3_O_4_ nanomaterial

Aluminum nitrate nonahydrate (3.7526 g) and manganese nitrate tetrahydrate (7.5634 g) were completely dissolved in 100.0 mL of distilled water, and a homogeneous solution at ambient temperature was obtained. The pH of the solution was adjusted to 10.50 with 0.2 M NaOH solution by drop-wise addition and constant vigorous stirring. Overnight, the solution was heated at 60–70 °C with constant stirring. The solution was then cooled to ambient temperature and the precipitate centrifuged at 2000 rpm. The supernatant solution was discarded and the precipitate preserved. The precipitate was washed with ethanol 1–2 times then allowed to dry at ambient temperature or in oven at 50–60 °C. The precipitate was ground and stored in clean, dry, and inert plastic vials.

### Proposed mechanism of nanomaterial growth

The growth of Al_2_O_3_ doped Mn_3_O_4_ nanomaterial may be elucidated on the basis of the following chemical reaction pathway:

1 $$ NaO{H}_{(aq)}\to N{a^{+}}_{(aq)}+O{H^{\_}}_{(aq)} $$

2 $$ Al{\left(N{O}_3\right)}_3\cdot 9{H}_2O+3NaO{H}_{(aq)}\to Al{(OH)_3}_{(aq)}+3N{a}^{+}+3N{O_3}^{\_}+9{H}_2O $$

3 $$ Mn{\left(N{O}_3\right)}_2\cdot 4{H}_2O+2NaO{H}_{(aq)}\to Mn{(OH)_2}_{(aq)}+2N{a}^{+}+2N{O_3}^{{}^{\_}}+4{H}_2O $$

4 $$ 3Mn{(OH)_2}_{(aq)}+2Al{(OH)_3}_{(aq)}+2NaO{H}_{(aq)}\to A{l}_2{O}_3\cdot \mathrm{M}{\mathrm{n}}_3{\mathrm{O}}_4+7{H}_2O+2N{a}^{+} $$

### Characterization

The surface morphology of the nanoparticles was studied using a EOL scanning electron microscope (JSM-7600F, Japan). Elemental analysis was carried out using EDS (Oxford). X-ray diffraction patterns (XRD) were taken with a computer-controlled X’Pert Explorer, PANalytical diffractometer. FT-IR spectra were recorded in the range of 400–4000 cm^−1^ on a Perkin Elmer (spectrum 100) FT-IR spectrometer. UV spectrum was recorded from 200 to 900 nm using UV-visible spectrophotometer (UV-2960, LABOMED INC.). An XPS survey scan was made by Thermo Scientific K-Alpha KA1066 spectrometer (Germany) in the range of 0–1350 eV.

### Photocatalytic degradation of dye

The photocatalytic activity of Al_2_O_3_ doped Mn_3_O_4_ multi-metal oxide nanoparticles was evaluated through degradation of BCB under visible light irradiation. The dye is stable under visible light irradiation in the absence of photocatalyst.

In photocatalytic degradation, two different 100.0 mL, 1 × 10^−4^ M of BCB dye solutions were taken in different beakers and adjusted to pH 5 and 10, respectively, by drop-wise addition of 0.2 M NaOH solution under vigorous stirring. Then, 0.1193 and 0.1132 g of catalyst was added to the reaction solutions and kept in the dark, for physical adsorption of dye on catalyst surface. It has been reported in previous photocatalytic studies on TiO_2_ that besides the light absorption capability and charge transportation, the adsorption of reactant is also a critical factor [7]. The sample solution was then irradiated under sunlight with constant stirring.

The dye solution of about 4–5 mL was pipetted out at regular intervals and the absorbance measured at *λ*_max_ = 595.0 nm by spectrophotometer (LABOMED INC.). Absorbances were measured at time intervals 0, 10, 20, 40, 60, 90, 120, 140, 160, 180, 200, 220, 240, 260, 280, and 300 min. The controlled experiments were also performed under visible light without catalyst to measure any possible direct photocatalysis of dye.

## Results and discussion

### Physiochemical characterization of Al_2_O_3_ doped Mn_3_O_4_ nanomaterial

#### Morphology study (FESEM)

**Fig. 1:**
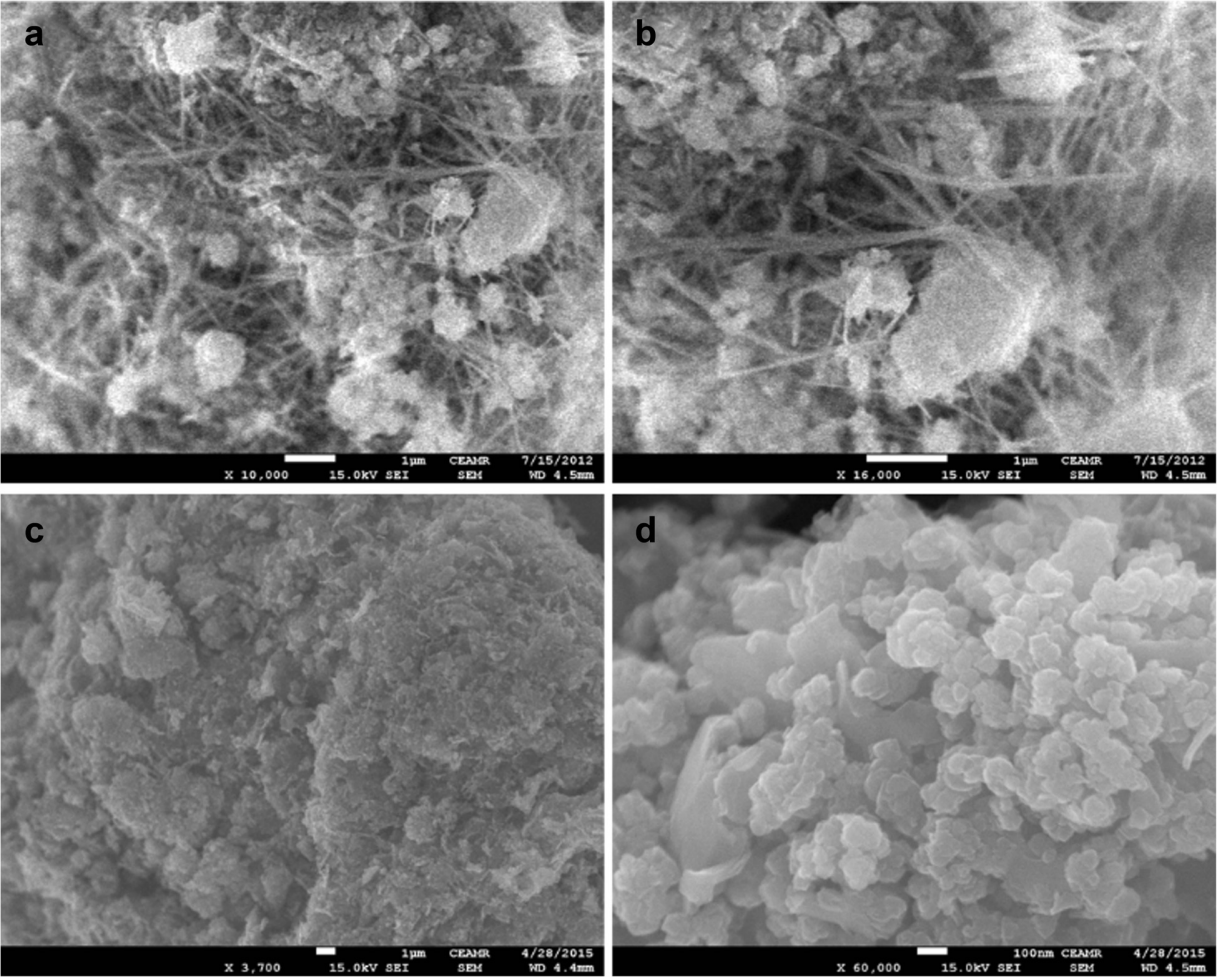
SEM image at low magnification (**a**) and high magnification (**b**) of Al_2_O_3_ doped Mn_3_O_4_ nanomaterial and Mn_3_O_4_ nanoparticles (**c, d**)

**Fig. 2:**
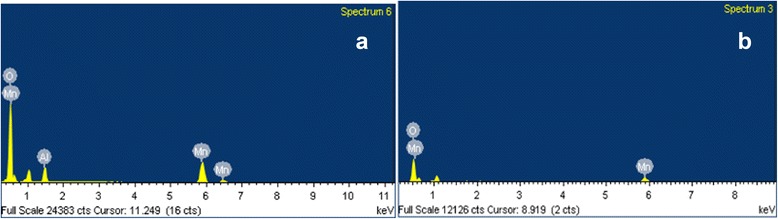
EDS spectrum of (**a**) Al_2_O_3_ doped Mn_3_O_4_ nanomaterial and (**b**) Mn_3_O_4_ nanoparticles

The morphology of ΝΜ was captured by FESEM images, which are shown in Fig. 1. Low and high magnification images of FESEM showed that the synthesized product is composed of nanowires and cumulative form of aggregated spherical-shaped nanoparticles. The FESEM images of synthesized pure Mn_3_O_4_ are shown in Fig. 1c, d which indicate that as-prepared nanomaterial is grown in the form of nanoparticles. To evaluate composition of the synthesized nanomaterial, EDS spectrum was analyzed and the data is depicted in Fig. 2a. EDS showed peaks related only to Al, Mn, and O with weight percentages of 8.24, 32.59, and 59.18, respectively, without any impurity peak, which confirmed that synthesized nanomaterial is composed of Al, Mn, and O. The EDS spectrum of pure Mn_3_O_4_ displayed peaks related to Mn and O (Fig. 2b).

#### Phase and compositional study (XRD)

**Fig. 3:**
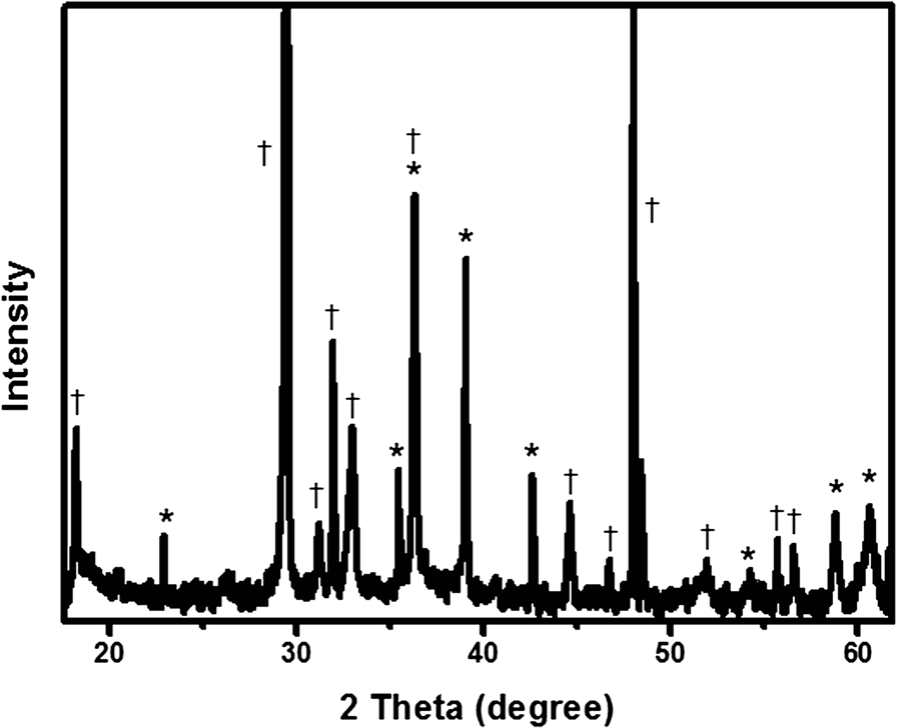
Powder XRD patterns of Al_2_O_3_ doped Mn_3_O_4_ nanomaterial

Crystal phase and crystallinity of ΝΜ was checked by XRD, which is shown in Fig. 3. The general feature of this XRD pattern and, in particular, the presence of strong and sharp peaks and the absence of diffraction halo indicate that the synthesized nanoparticles are fully crystalline without amorphous or crystalline-amorphous phase. XRD displayed sharp peaks at 2*θ* = 18.2, 23.0, 29.5, 31.1, 31.9, 33.1, 35.4, 36.5, 39.0, 42.6, 44.8, 48.3, 52.0, 54.2, 55.7, 56.7, and 58.8. All these peaks correspond to the Al_2_O_3_ and tetragonal Mn_3_O_4_ phases. The diffraction peaks at 23.0, 36.5, 39.0, 42.6, 54.2, 56.7, and 61.5 (marked with an asterisk symbols) indicated the presence of α-Al_2_O_3_ [10, 11]. The characteristic peaks marked with dagger symbols are indexed to (101), (112), (200), (103), (211), (004), (220), (105), (321), and (224) and confirmed the presence of tetragonal Mn_3_O_4_ phase. The peaks obtained for Mn_3_O_4_ are in good agreement with the literature (JCPDS 44-0992, JCPDS 24-0734) [12, 13].

#### X-ray photoelectron spectroscopy (XPS)

**Fig. 4:**
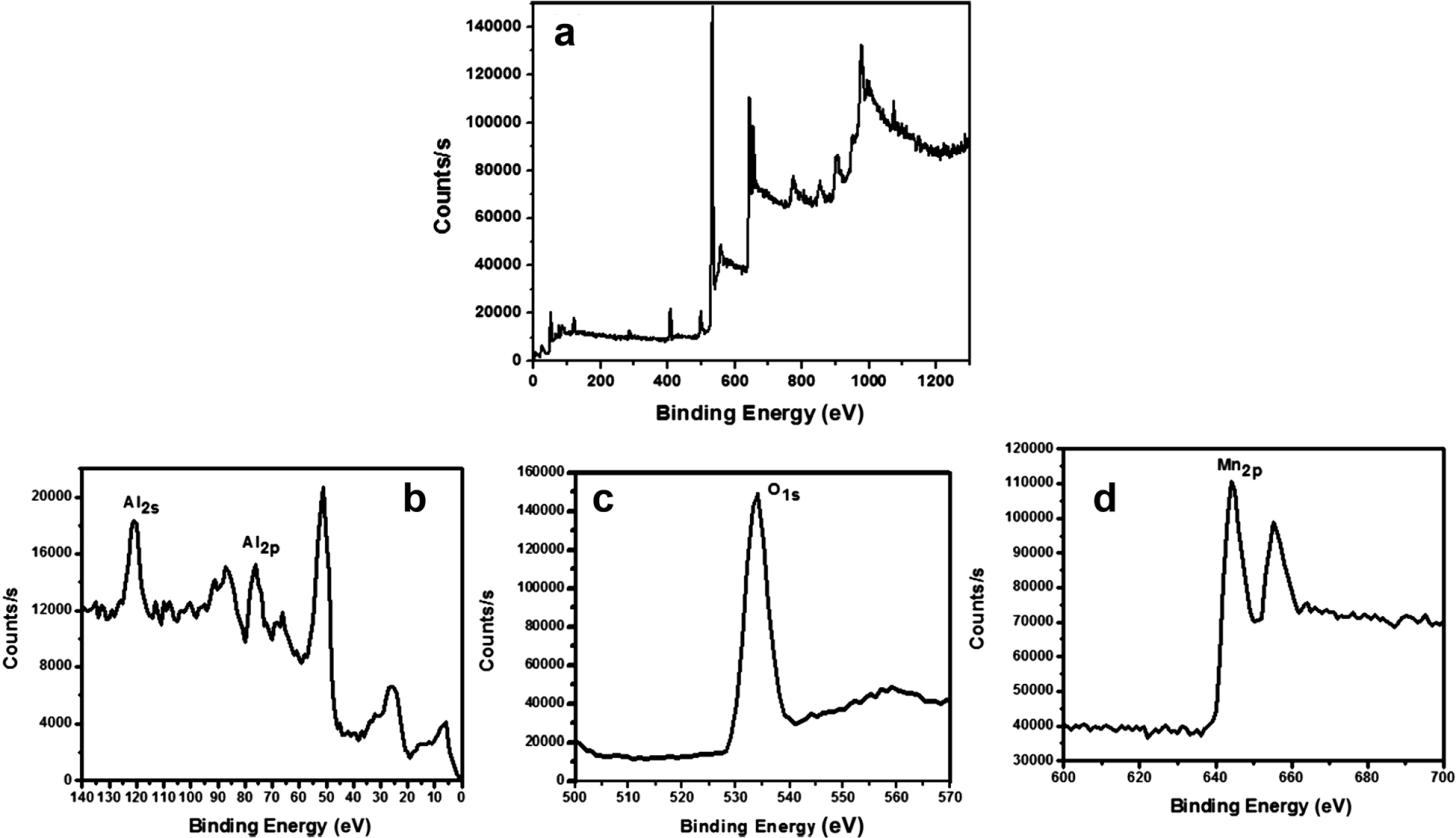
XPS spectra of Al_2_O_3_ doped Mn_3_O_4_ nanomaterial

**Fig. 5:**
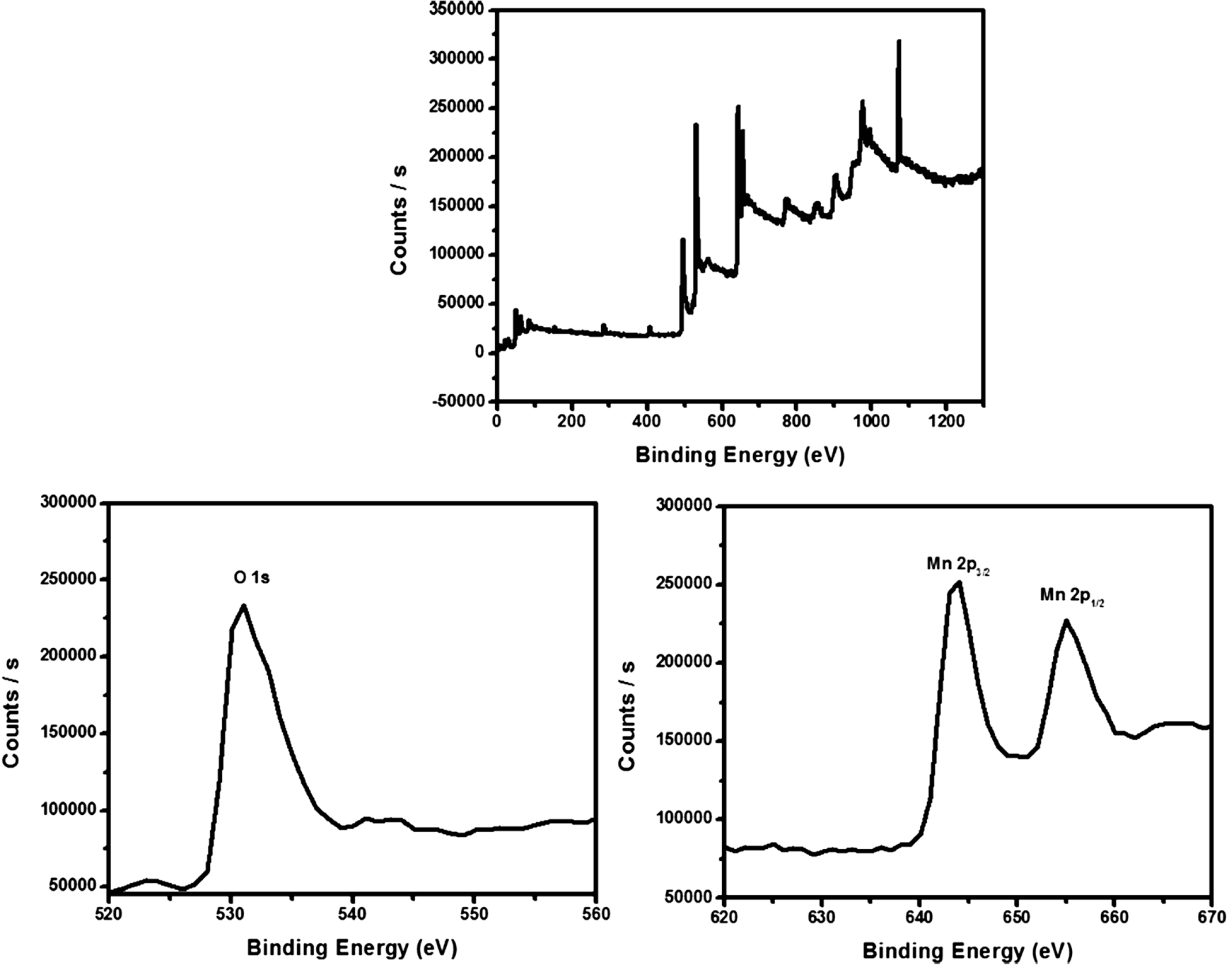
XPS spectra of Mn_3_O_4_ nanoparticles

The bonding configuration and compositional analysis of as-grown Al_2_O_3_ doped Mn_3_O_4_ nanomaterial was carried out by X-ray photoelectron spectroscopy. Figure 4 shows the XPS survey scan of NM. There are four important characterized peaks of elastic collision of electron with the surface of solid material. The XPS spectrum corresponding to peaks of Mn 2p_3/2_ and Mn 2p_1/2_ has binding energy (BE) values of 644.26 and 655.10 eV, respectively (Fig. 4a). These BE values for Mn_3_O_4_ are in agreement with values reported in the literature [14]. The spin-orbit coupling, measured by calculating the difference between the BE value of Mn 2p_3/2_ and Mn 2p_1/2_ levels, was found to be 10.84 eV, which is comparable with the reported value of Mn_3_O_4_ [14]. The occurrence of the peak corresponding to the binding energy of O 1s (534.05 eV) is shown in Fig. 4b. The binding energies of the Al 2p and Al 2s are located at 75.89 and 120.95 eV, as shown in Fig. 4c. This study confirmed the presence of Al_2_O_3_ [15]. The XPS spectrum of pure Mn_3_O_4_ exhibited peaks for Mn 2p_3/2_, Mn 2p_1/2_, and O 1s at binding energy (BE) values of 644.10, 655.07, and 531.04 eV, respectively (Fig. 5). This confirms the formation of Mn_3_O_4_.

#### FT-IR analysis

**Fig. 6:**
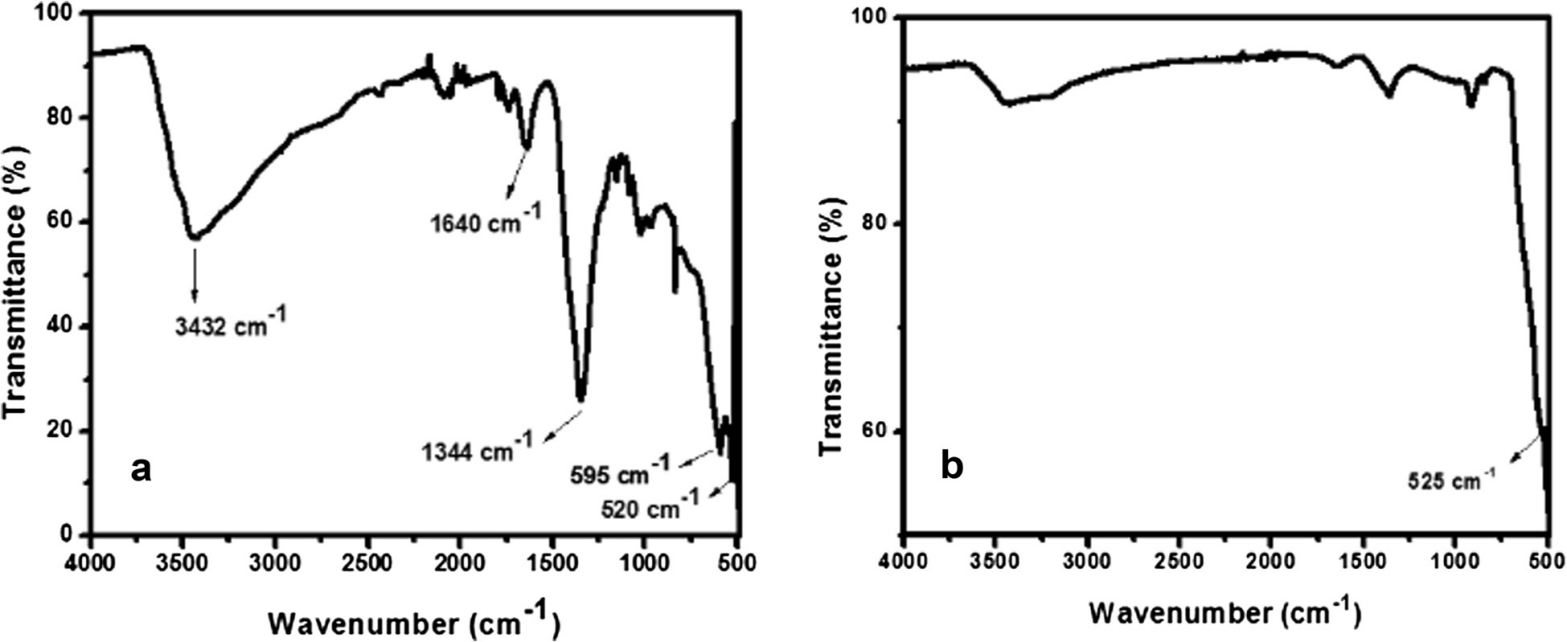
FT-IR spectra of **a** Al_2_O_3_ doped Mn_3_O_4_ nanomaterial and **b** Mn_3_O_4_ nanoparticles

FT-IR analysis was performed to establish the vibrational transition of various bonds present in our as-grown nanomaterial, which is shown in Fig. 6a. It illustrated that the qualitative peaks of M-O and O-M-O vibration stretching occurred at 520 and 595 cm^−1^, respectively. Furthermore, spectrum elaborated sharp peaks corresponding to 1344 cm^−1^ can be attributed to the adsorption of CO_2_ or CO_3_^2−^, which is in line with the literature [16–18]. There are also peaks at 1640 and 3432 cm^−1^, corresponding to O-H bending and O-H stretching vibrations, which confirmed the presence of moisture adsorbed from the atmosphere by the as-prepared nanomaterial. The FTIR spectrum of pure Mn_3_O_4_ showed an intense peak at 525 cm^−1^ which is responsible for M-O stretching vibration (Fig. 6b).

#### Photo-absorption properties and band gap energy

**Fig. 7:**
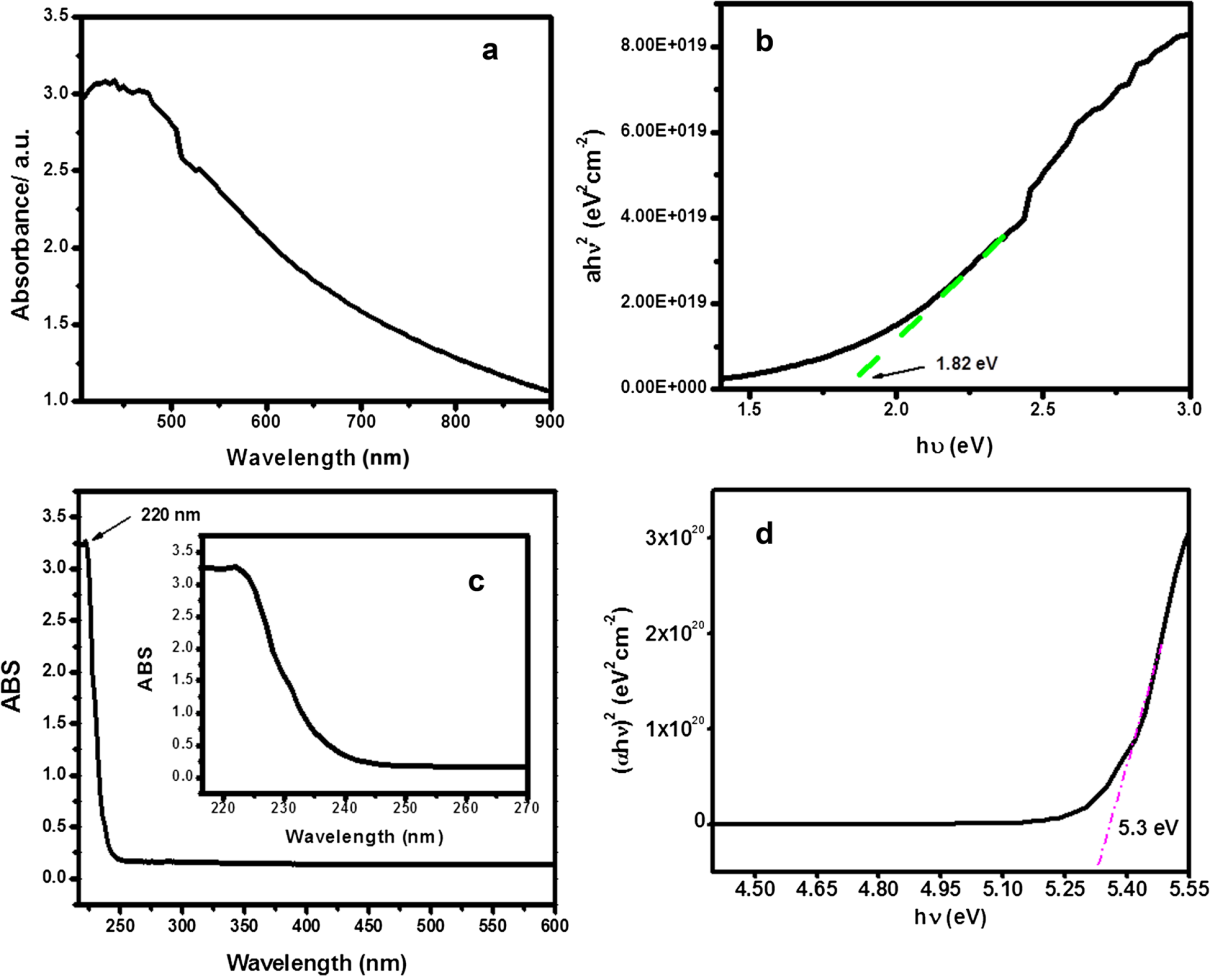
UV-visible spectrum of Al_2_O_3_ doped Mn_3_O_4_ nanomaterial (**a**), *(*α*h*ν*)* ^*2*^ vs. *h*ν plot of Al_2_O_3_ doped Mn_3_O_4_ nanomaterial (**b**), UV-visible spectrum of Mn_3_O_4_ nanoparticles (**c**), and *(*α*h*ν*)* ^*2*^ vs. *h*ν plot of Mn_3_O_4_ nanoparticles (**d**)

Since photocatalytic reaction accounts for the electronic structure of the photocatalyst, there are two critical factors that influence photocatalysis: light absorption by the material and migration of light-induced electron holes. Theoretically, photon absorption is proportional to the mobility of electron-hole pairs, which in turn determines the probability of the electron and hole reaching the reaction sites on the surface of the photocatalyst. Figure 7a illustrates the strong absorption all over the visible electromagnetic radiation which is attributed to valence-conduction band transition (i.e., charge separation). The band gap energy *E*_*g*_ of Al_2_O_3_ doped Mn_3_O_4_ materials is found to be around 1.82 eV from the tangent drawn at the linear plateau of the curve (*αhν*)^2^ vs. *hv* (Fig. 7b) [19–21]. Pure Mn_3_O_4_ illustrates a strong absorption band at 220 nm (Fig. 7c) and the band gap energy *E*_*g*_ is found to be 5.30 eV (Fig. 7d).

**Fig. 8:**
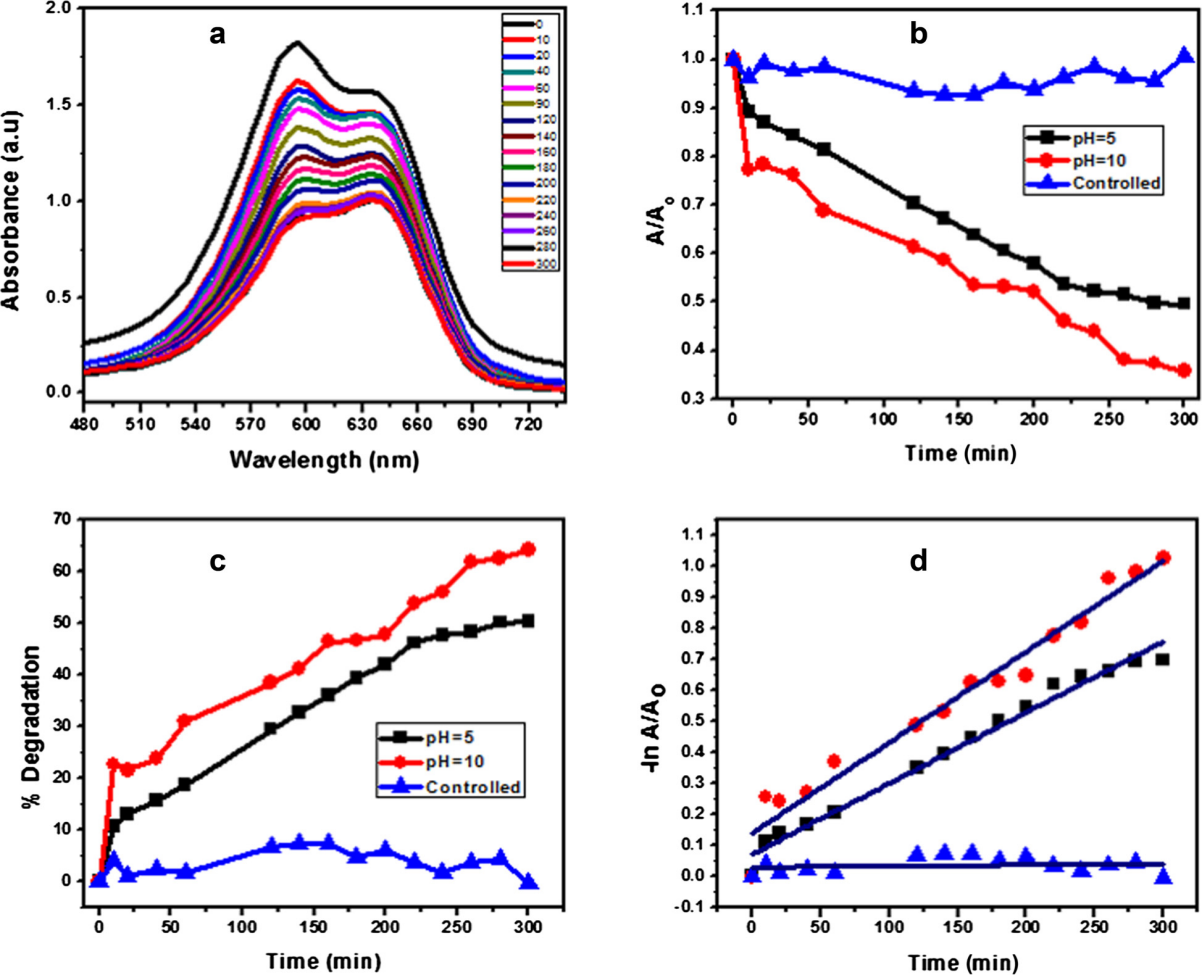
Typical plot for (**a**) change in the absorption spectrum of BCB, (**b**) comparison of change in absorbance vs. irradiation time for BCB, (**c**) comparison of % degradation vs. irradiation time for BCB, and (**d**) linear regression analysis between ‘ln’ of change in absorbance vs. irradiation time for BCB

### Photocatalytic activities

#### Effect of pH

The effect of pH on the visible light photocatalytic degradation of basic dye BCB was studied in pH range 5–10 at metal oxide nanomaterial and 1 × 10^−4^ M dye concentration. The results showed that the rate of decolorization increased with the increase in pH from 5 to 10 (Fig. 8b). The photocatalytic performance of the metal oxide was attributed to the surface electrical properties, which facilitate the dye adsorption. It is beneficial for the promotion of visible light generated charge carrier, i.e., electron, to the surface, which leads to the formation of hydroxide radical. Moreover, the pH of the dye solution has a substantial influence on the photocatalytic degradation process, so pH 10 is recommended for basic dyes.

#### Control experiments and photocatalysis

In this study, three sets of photocatalytic reaction were performed using ΝΜ. First, the experiment without catalyst under visible light irradiation resulted in a small amount of degradation, indicating photolysis reaction. Second, a controlled experiment was performed under dark conditions for 30 min, indicating the physical adsorption of dye on the surface of the photocatalyst. This indicates that the equilibrium time for the dye is reached within 30 min. Finally, photocatalytic degradation of well-stirred dye (BCB) solution was carried out in the presence of the photocatalyst under visible light irradiation. Metal oxide showed efficient activity for degradation of BCB dye at different pH under solar light irradiation (Fig. 8c). Furthermore, the absorption spectrum of BCB dye, presented in Fig. 8a, reflected 50–65 % decolorization after 5 h (300 min).

#### Kinetic study of dye

The Langmuir–Hinshelwood (L-H) model was successfully applied to the visible light photocatalytic degradation of organic dye and represented the best linear relationship between photocatalytic degradation rate and initial concentration of organic pollutant [20]. The rate expression is given by Eq. 5.

5 $$ r=-dC/dt\kern0.5em ={K}_rKC={K}_{\mathrm{app}}C $$

where *r* is the degradation rate of dyes (organic pollutant), *K*_*r*_ is the reaction rate constant, *K* is the equilibrium constant, and *C* is the reactant concentration. When *C* was very small, *KC* was negligible, so that Eq. 5 became the first-order kinetics. Setting Eq. 5 under initial conditions of photocatalytic procedure, (*t* = 0, *C = C*_*o*_), it became Eq. 6.

6 $$ ln\left(C/{C}_o\right)={K}_{\mathrm{app}}t $$

*C*_*o*_ was the initial concentration of dye, and *C* was the concentration at time “*t*”. Using Eq. 6, we calculated the apparent rate constant from the gradient of the graph of ln(*C/C*_*o*_), corresponding to ln(*A*/*A*_*o*_) vs. irradiation time.

The apparent rate constant of photocatalytic activity was independent of adsorption and the concentration of the dye remaining in the solution. Figure 8d illustrates that the degradation of BCB dye followed the first-order kinetics as the plot of the variations of ln(*C/C*_*o*_) as a function of irradiation time showed linearity. Table 1 shows the corresponding first-order rate constant *K*_*app*_, evaluated from the slopes of the linear plot, *t*_*1/2*_ parameters (time required to degrade half of the initial concentration of dye) and regression relative coefficient values.

**Table 1:** Pseudo first-order kinetic study for BCB dye with and without Al_2_O_3_ doped Mn_3_O_4_ nanomaterial

pH	*K*_app_ (min^−1^)	Rate of decolorization	*R*	*R* ^2^	*t*_1/2_ (min)	*t*_1/2_ (years)
With catalyst						
5	0.00229	2.2923E^−07^	0.9918	0.9836	302.3129	0.00058
10	0.00293	2.9340E^−07^	0.9826	0.9654	236.1941	0.00045
Without catalyst						
5	3.81149E^−05^	0.0381E^−07^	0.1478	0.0218	18181.8493	0.03459

#### Proposed mechanism and discussion of visible light photocatalytic reaction using oxides

**Fig. 9:**
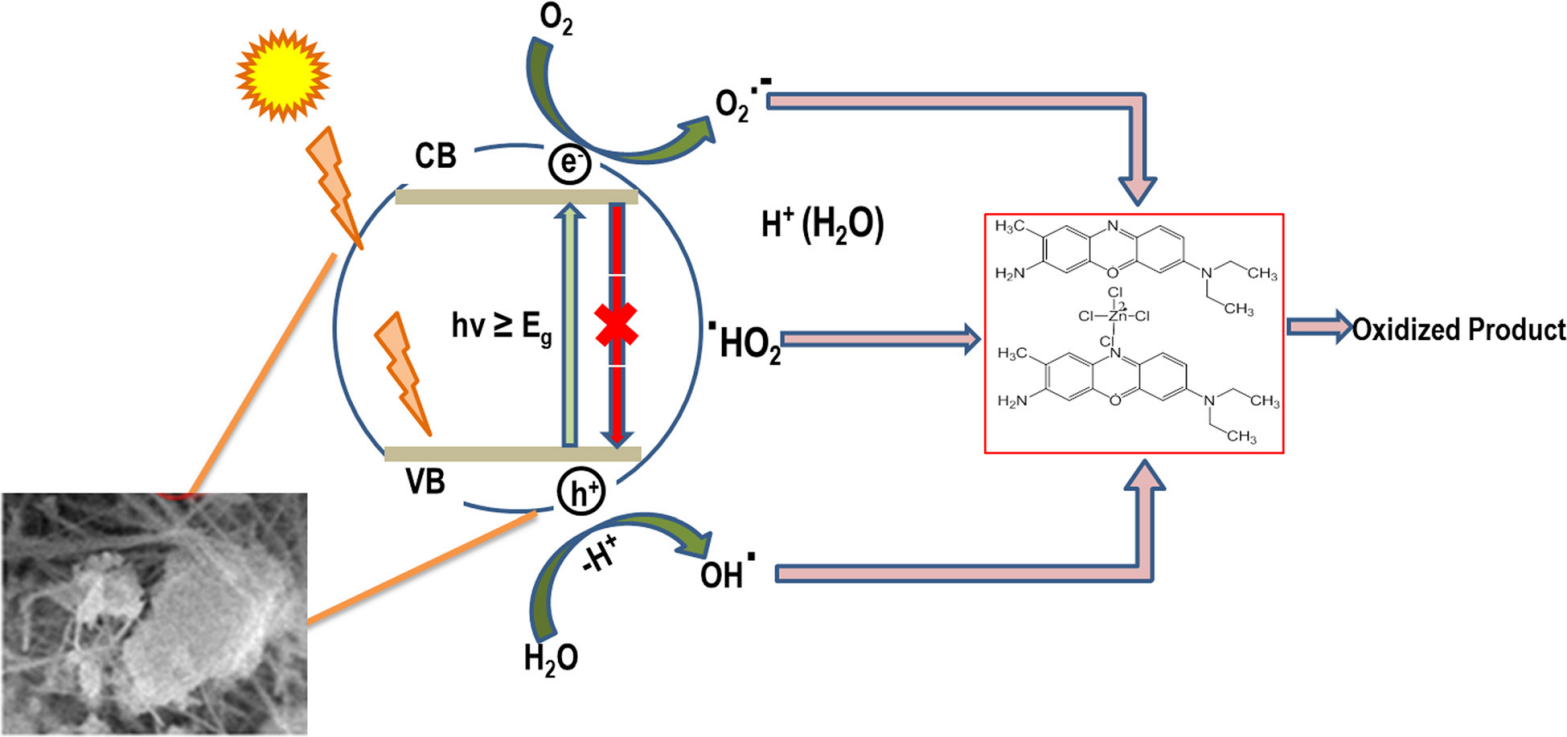
Schematic view of photo-degradation using Al_2_O_3_ doped Mn_3_O_4_ nanomaterial

The basic mechanism of photocatalytic degradation of organic dye using wide spectrum of solar light, i.e., visible light, is shown in Fig. 9. Introducing ultra-band gap visible light energy to the NM causes the valence band electron to be promoted to conduction band, leaving behind charge separation (Eq. 5). This electron-hole pair drifts to the NM surface to participate in oxidation-reduction reaction. A portion of the photo-generated electrons would recombine with holes in the VB, while others are transferred to the surface, where the surrounding molecular oxygen can scavenge electrons from NM’s conduction band and transform them into superoxide anion radical (O_2_^•−^) (Eq. 6), since the conduction band of NM is nearly iso-energetic with the reduction potential of oxygen. Later, the superoxide radical reacts with a proton, forming hydrogen peroxide (H_2_O_2_) followed by hydroxyl radical (^•^OH) (Eq. 7). Furthermore, the hole (h^+^) in VB generates hydroxyl radicals (^•^OH) from water solvent (Eq. 8).

Continued attacks of O_2_^•−^ and ^•^OH radicals on pollutant species (organic dye) lead to the degradation (oxidation) of the dye molecule (Eq. 9). We can also supply external hydrogen peroxide to the reaction system, which have a significant potential to produce the hydroxyl radicals. It is essential for an efficient photocatalytic process to have the charge carriers separated as far as possible. Moreover, the dye molecule, BCB, adsorbed on nanomaterial may be stimulated to excited state under visible light irradiation. Subsequently, photo-excited dye electrons may be inoculated into the conduction band of nanomaterial via photosensitization. Additionally, dye molecules are degraded (oxidation) by the photo-generated holes in the valence band of nanomaterials [21, 19].

Based on the discussion above, a possible mechanism for the photocatalytic degradation of organic dye BCB over NM can be proposed, as illustrated by Eqs. 7, 8, 9, 10 and 11.

7 $$ A{l}_2{O}_3/M{n}_3{O}_4+hv\to \kern1em A{l}_2{O}_3/M{n}_3{O}_4\kern0.5em \left({e_{CB}}^{-}+{h_{VB}}^{+}\right) $$

8 $$ A{l}_2{O}_3/M{n}_3{O}_4\kern0.5em \left({e_{CB}}^{-}\right)+{O}_2\to \kern1em A{l}_2{O}_3/M{n}_3{O}_4+{O_2}^{-} $$

9 $$ {O}_2{{}^{\cdot}}^{{}^{{}_{{}^{\_}}}}+{H}^{+}\left({H}_2O\right)\to {H}_2{O}_2\to OH\cdot $$

10 $$ A{l}_2{O}_3/M{n}_3{O}_4\kern0.5em \left({h_{VB}}^{+}\right)+{H}_2O\to A{1}_2{O}_3/M{n}_3{O}_4+OH\cdot $$

11 $$ OH\cdot +BCB\to Oxidized\kern0.5em  products $$

## Conclusions

A simple low-temperature and low-cost solar photocatalyst based on Al_2_O_3_ doped Mn_3_O_4_ nanomaterial has been developed for the degradation of BCB. Singlet oxygen (O_2_^•−^) and hydroxyl (^•^OH) radicals generated by oxidation and reduction reaction of O_2_ and H_2_O, respectively, are primarily responsible for photocatalyzing the degradation of organic pollutant BCB under the irradiation of solar light. The decay of BCB follows the pseudo first-order kinetics which satisfied the Langmuir-Hinshelwood (L-H) kinetic model. This research represents an important advance in the synthesis of novel co-doped oxides for the photocatalytic degradation of organic pollutant under visible light irradiation for industrial effluent.
